# Biotics-based modulation of gastric precancerous states: microbial, metabolic, and epithelial mechanisms

**DOI:** 10.3389/fimmu.2026.1927527

**Published:** 2026-10-07

**Authors:** Xi Li, Jian Ma, Gaoyan Shen, Rui Liu, Yun Wang, Jiajun Zhang, Yinling Guo, Xiaoying Hao

**Affiliations:** 1 School of Basic Medical Sciences, Chengdu University of Traditional Chinese Medicine, Chengdu, China; 2 Affiliated Zigong Hospital of Chengdu University of Traditional Chinese Medicine, Zigong, China

**Keywords:** engineered probiotics, gastric precancerous lesions, *Helicobacter pylori*, mucosal immunity, postbiotics, probiotics

## Abstract

Gastric precancerous states are important intermediate stages in gastric carcinogenesis, yet strategies that directly modify their persistent biological drivers remain limited. Current management mainly includes *Helicobacter pylori* eradication, endoscopic surveillance, and mucosal protective strategies. Biotics, including probiotics, synbiotics, postbiotics, and next-generation microbial products, have been investigated as potential strategies to modulate persistent microbial, metabolic, inflammatory, and barrier alterations. However, existing evidence derives from studies of *H. pylori*-associated gastritis and gastric precancerous lesions, as well as related gastric cancer and gastrointestinal models, making it important to distinguish direct evidence from findings in related disease contexts. This review examines evidence for biotic modulation of microbial homeostasis, metabolic and postbiotic signaling, immune and barrier function, and host–microbiota interactions across anatomical compartments during gastric precancerous progression. Current evidence is concentrated largely in *H. pylori*-associated gastritis, where biotics have been reported to influence microbial composition, inflammatory signaling, metabolic profiles, and barrier function, whereas direct evidence in established atrophy, intestinal metaplasia, and dysplasia remains limited. These findings support further investigation of biotics as strategies for risk modification and homeostatic support rather than as substitutes for established clinical management. Future studies will require better standardization of strains, formulations, dosage, and treatment duration, together with clearly defined histological outcomes and long-term clinical endpoints.

## 1 Introduction

Gastric cancer remains a major global health burden and is among the leading causes of cancer-related mortality worldwide. Gastric carcinogenesis is a multistep process characterized by chronic inflammation, mucosal injury, and aberrant repair. Following the Correa cascade, this progression advances from chronic gastritis and glandular atrophy to gastric intestinal metaplasia (GIM), dysplasia, and ultimately, intestinal-type gastric cancer (1–3). Current guidelines recognize atrophic gastritis, intestinal metaplasia, and dysplasia as established gastric premalignant conditions or lesions. In this review, we take a broader mechanistic view by including *H. pylori*-associated chronic non-atrophic gastritis as the inflammatory first step in the Correa cascade, rather than as a premalignant lesion itself (4–7). Notably, gastric precancerous states follow a prolonged and dynamic course, providing a potential window for early intervention and risk modification (8). However, the clinical management of gastric precancerous lesions (GPLs) remains largely dependent on *Helicobacter pylori* eradication and endoscopic surveillance. Mechanism-based interventions that directly target the biological drivers of disease progression are still limited, particularly during the stages of atrophy and intestinal metaplasia (IM). Pathogen eradication alone does not fully eliminate the long-term risk of gastric carcinogenesis (5, 9).

Beyond clinical management, molecular and single-cell studies have shown that GPLs involve persistent epithelial and microenvironmental alterations rather than transient mucosal inflammation alone. At this stage, the gastric epithelium exhibits altered cellular differentiation and lineage plasticity (10, 11). Single-cell analysis show that precancerous alterations occur alongside epithelial identity instability and progressive remodeling of the tumor-promoting microenvironment (12, 13). Even after *H. pylori* eradication, epigenetic alterations may partially reverse in non-metaplastic mucosa but tend to persist in the context of IM, suggesting that GPLs represent a risk state shaped by field effects and epigenetic memory (14–16).

This view of GPL as a “risk state” is also reflected in current clinical practice. The management of individuals with atrophic gastritis and IM should be guided by risk stratification and ongoing surveillance, rather than equating risk control solely with *H. pylori* eradication (4, 17). It is also supported by microbiome studies showing that, under hypochlorhydric or achlorhydric conditions caused by atrophy, non-*H. pylori* bacteria may expand and intestinal microbes may colonize the stomach, thereby contributing to persistent inflammation, metabolic disturbance, and epithelial injury (18, 19). These observations provide a rationale for investigating whether biotic interventions can modulate microbial, inflammatory, metabolic, and barrier alterations associated with gastric precancerous states (20).

Therefore, a focused synthesis is needed to clarify the potential role and evidentiary boundaries of biotic interventions across different stages of gastric precancerous progression. Current evidence is concentrated largely in *H. pylori*-associated gastritis, with reported effects involving microbial composition, inflammatory signaling, metabolic profiles, and barrier function. Direct evidence in established atrophy, intestinal metaplasia, and dysplasia remains substantially more limited, particularly for histological outcomes.

Consequently, the potential role of biotics should be considered according to disease stage, as strategies for risk modification and homeostatic support rather than as curative interventions that reverse established structural lesions or eliminate cancer risk. This review therefore synthesizes current evidence on the effects, mechanisms, and evidentiary boundaries of biotic interventions across different stages of gastric precancerous progression. The progression of gastric precancerous states and the major forms of biotic interventions are summarized in **Figure 1**.

**Figure 1:**
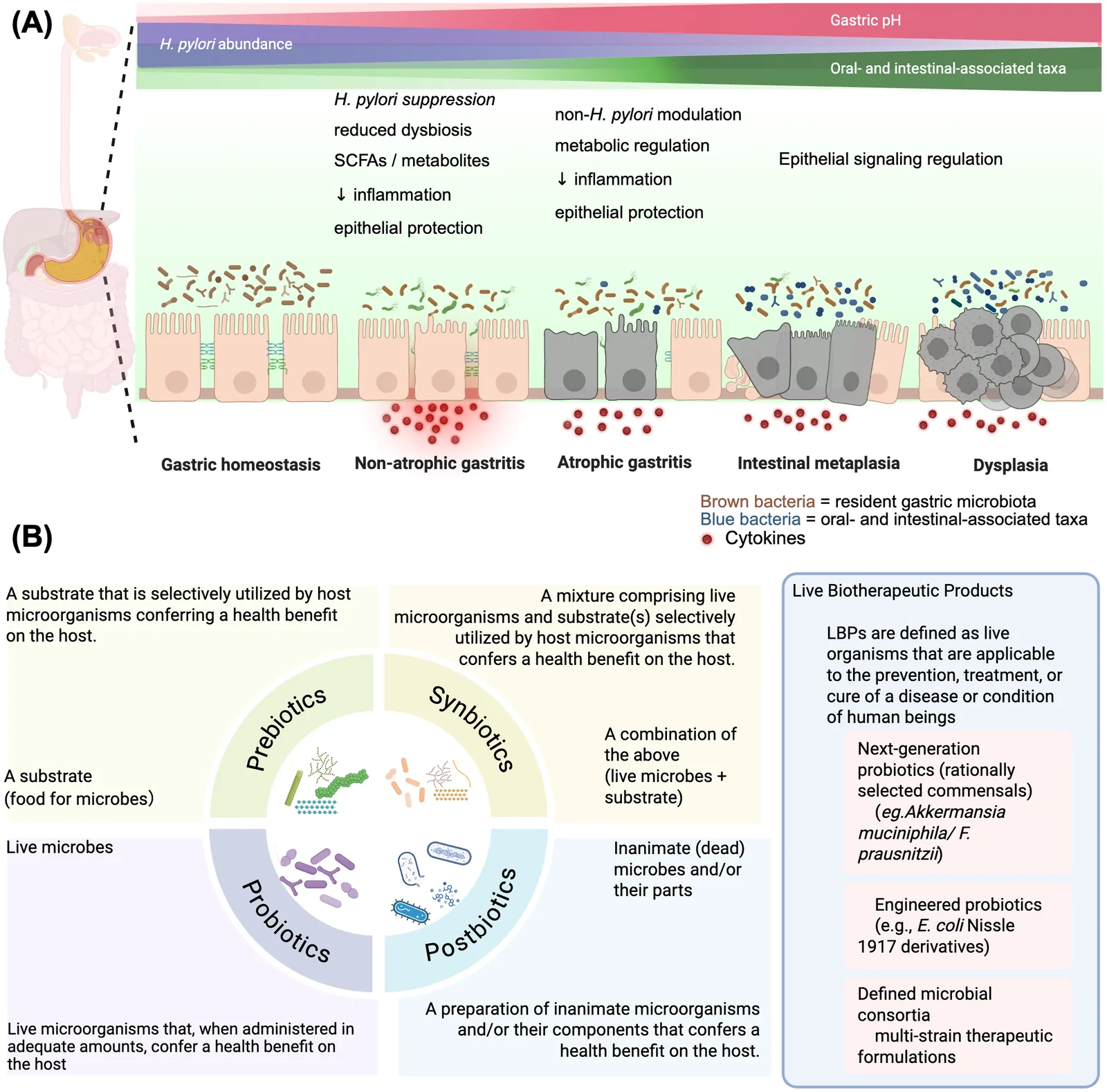
Stage-dependent microbial alterations and positioning of biotic interventions during gastric precancerous progression, and major categories of biotic interventions. **(A)** Stage-dependent changes in the gastric microenvironment during progression from gastric homeostasis to non-atrophic gastritis, atrophic gastritis, intestinal metaplasia, and dysplasia. The schematic depicts a progressive decline in *H. pylori* abundance, an increase in gastric pH, and increased abundance of oral- and intestinal-associated taxa. The proposed roles of biotic interventions are correspondingly stage dependent: evidence at the gastritis stage primarily supports *H. pylori* suppression, reduction of dysbiosis, changes in SCFA levels, attenuation of inflammation, and epithelial protection; evidence in atrophic gastritis is more limited and mainly concerns non-*H. pylori* microbial modulation, metabolic regulation, inflammation, and epithelial protection; evidence in intestinal metaplasia is sparse and involves modulation of epithelial signaling; direct evidence in dysplasia remains lacking. **(B)** Major categories of biotics, including probiotics, prebiotics, synbiotics, and postbiotics, together with emerging microbiome-based therapeutic approaches such as live biotherapeutic products (LBPs), next-generation probiotics, engineered probiotics, and defined microbial consortia. Created with BioRender.com (https://BioRender.com/gc0ueqk).

## 2 Probiotics, synbiotics, and postbiotics: functional evolution and mechanistic basis

### 2.1 Definition and functional evolution

The understanding of probiotic interventions is not limited to the notion of live microbial supplementation. Rather, the evolution of this concept reflects a deepening understanding of the mechanisms governing host–microbiome interactions (21). The term “probiotic” was first introduced in 1965 by Lilly and Stillwell to describe growth-promoting factors produced by certain protozoa during the logarithmic phase, which extended the growth of other protozoa by approximately 50% (22). At that time, the term referred to microorganism-derived substances rather than live microorganisms per se. As research shifted from protozoan culture systems to microbe–host interactions and clinical applications, the definition of probiotics evolved from growth-promoting factors to “live microorganisms that, when administered in adequate amounts, confer a health benefit on the host,” emphasizing viability and safety. In 2014, the International Scientific Association for Probiotics and Prebiotics (ISAPP) published a consensus statement that defined probiotics as “live microorganisms that, when administered in adequate amounts, confer a health benefit on the host” (23). This refinement shifted the concept of probiotics from products merely containing microorganisms to functionally defined microbial interventions grounded in demonstrated health benefits. Accordingly, their value lies not in increasing microbial abundance, but in delivering defined health benefits through strain-specific effects.

As research expanded from individual microbial activities to broader host–microbiome interactions, ISAPP updated the definition of synbiotics in 2020, describing them as mixtures of live microorganisms and selectively utilized substrates that confer a health benefit (24). In 2021, postbiotics were defined as preparations of inanimate microorganisms and/or their components that confer a health benefit on the host (25–28). Together, these revisions formalized a shift from product composition to function-based definitions.

### 2.2 Next-generation probiotics

As understanding of gut microbial function has deepened, a new class of microorganisms distinct from traditional *Lactobacillus* and *Bifidobacterium* has emerged, termed next-generation probiotics (NGPs). NGPs are members of the core human gut microbiota that are associated with specific host phenotypes or disease states, rather than defined by a long history of safe food use (29). The U.S. Food and Drug Administration (FDA) defines a live biotherapeutic product (LBP) as a biological product containing live organisms intended for the prevention, treatment, or cure of disease in humans, excluding vaccines (30). The clinical development of LBPs requires pharmaceutical-grade quality control and investigational new drug regulatory pathways (31). This regulatory distinction represents a transition from nutritional supplementation to disease-targeted microbial therapeutics.

Among natural NGPs, several core human commensals exhibit functional capacities beyond those of traditional lactic acid bacteria. *Akkermansia muciniphila* is widely regarded as a representative NGP, characterized by its interaction with the mucus layer and its influence on host metabolic signaling, with beneficial effects in metabolic and inflammatory disorders (32). *Faecalibacterium prausnitzii* has attracted attention for its anti-inflammatory and metabolic effects; however, its clinical application remains limited by cultivation and delivery challenges (33). The genus *Bacteroides*, which possesses modular polysaccharide utilization loci (PULs), immunomodulatory molecules such as polysaccharide A, and genomic plasticity, has been proposed as a platform linking natural NGPs to engineered microbial therapeutic strategies (34, 35).

In contrast to NGPs, engineered probiotics are developed using synthetic biology approaches, including gene editing and synthetic circuit design. Such strategies are designed to improve functional precision and consistency, enabling controlled sensing and response to specific biological cues. In this way, engineered probiotics provide a conceptual link between microbiome-based interventions and precision medicine (36, 37).

From an engineering perspective, the development of engineered probiotics generally follows a three-tiered architecture consisting of chassis selection, functional module assembly, and safety and delivery control. Chassis strains are typically derived from microorganisms with established safety records or prior clinical use. Among these, *Escherichia coli* Nissle 1917 has become a widely used platform owing to its intestinal colonization capacity, genetic tractability, and established regulatory profile (38). Functional modules are designed to target defined biological processes, including anti-inflammatory activity, immunomodulation, and pathogen suppression. To improve control *in vivo* while ensuring biosafety, strategies such as synthetic genetic circuits, biocontainment switches such as kill switches, and biomaterial-based encapsulation are commonly employed (39–41). The development of engineered probiotics for phenylketonuria (PKU) provides an illustrative example of clinical translation. Synthetic live biotherapeutics SYNB1618 and SYNB1934, built on the *E. coli* Nissle 1917 chassis and engineered to degrade phenylalanine in the gut, function as intestinal metabolic scavengers (42, 43). These candidates have progressed from preclinical development to phase II clinical evaluation, providing clinical evidence for the feasibility of engineered microbial therapeutics (44).

The application of engineered probiotics in gastric diseases faces several challenges, including limited gastric residence time and the niche-specific colonization of pathogens within the gastric mucosa. To target *H. pylori*, an engineered probiotic based on the food-grade bacterium *Lactococcus lactis* has been developed to secrete a guided antimicrobial peptide. By incorporating an *H. pylori*-specific recognition domain, this system enables targeted inhibition of the pathogen while minimizing nonspecific disruption of commensal microbiota (45). This study provides a proof-of-concept for gastric-targeted engineered probiotics, highlighting the potential of engineered microbes for pathogen-specific intervention in the gastric microenvironment. Nevertheless, despite these advances, most engineered probiotic strategies remain in the early stages of development, and their clinical application in gastric precancerous lesions has yet to be established.

### 2.3 Material-mediated precision delivery of biotics

Unlike traditional small-molecule or protein therapeutics, the delivery of LBPs faces distinct challenges, including targeting, controlled release, and maintenance of biological activity *in vivo*. Biomaterials such as hydrogels, nanoparticles, and scaffolds have emerged as important tools to address these challenges and support the clinical translation of LBPs (46).

The performance of biomaterial-based delivery systems is fundamentally determined by their material properties. Existing material-based delivery systems can be categorized into three functional strategies: protection, localization and retention, and stimulus-responsive release (47). Probiotic delivery has largely relied on polymers such as alginate and chitosan as carriers. Microencapsulation systems are typically constructed using techniques such as physical entrapment and ionic gelation, primarily to enhance probiotic viability during gastrointestinal transit (48, 49). However, physical encapsulation alone is insufficient for effective therapeutic intervention in complex microenvironments. This limitation has driven the development of smart biomaterial-based delivery systems that increasingly incorporate stimulus-responsive and functionally integrated designs, such as pH-responsive release, hydrogel-based microspheres, and multilayer encapsulation (50–52).

For gastric applications, biomaterial engineering has emerged as a key strategy to improve the delivery and retention of live microorganisms. A representative approach involves loading *Lactobacillus reuteri* into a transformable hydrogel system formulated as a lyophilized powder. Upon oral administration, it rapidly forms a hydrogel in the gastric environment. In *H. pylori*-infected mice, this system enhanced bacterial survival and gastric retention while enabling stimulus-responsive release in the inflammatory gastric microenvironment. The system also showed antioxidant and mucosal repair effects, inhibited *H. pylori*, and preserved gut microbiota homeostasis (53). The “3R” strategy (remove–remodel–repair) integrates pathogen clearance, inflammation modulation, and mucosal repair through biomaterial-based interventions (54). This concept fits well with the pathological features of GPL, including persistent low-grade inflammation and progressive mucosal remodeling.

Overall, biomaterials are not merely passive carriers for live microorganisms but serve as functional platforms that enhance microbial survival and enable controlled therapeutic delivery. By tailoring their physicochemical properties, these systems support targeted and responsive interventions in complex gastric environments. However, their application in gastric precancerous lesions remains largely preclinical, and their clinical efficacy has yet to be established.

## 3 Gastric precancerous states: mechanisms of disease initiation and progression

Compared with early *H. pylori*-associated gastritis, GPL is increasingly shaped by concurrent dysbiosis, chronic inflammation, and metabolic reprogramming. These processes form a self-reinforcing loop that promotes progression from atrophy to metaplasia and dysplasia.

### 3.1 Pathological progression and microbial drivers

Intestinal-type gastric cancer (Lauren classification) generally follows the Correa cascade, characterized by chronic inflammation-driven progression from atrophy through lineage reprogramming to preneoplastic lesions. The sequence comprises superficial gastritis, glandular atrophy, GIM, and dysplasia (1, 55). Progression is non-linear and sustained by persistent inflammation, mucosal microbiota disruption, and epithelial lineage remodeling. GIM represents a key inflection point in this sequence, reflecting lineage reprogramming at the stem/progenitor cell level rather than simple phenotypic replacement. Incomplete GIM (sulfomucin-rich type III) shows increased genetic and epigenetic instability and is most strongly associated with preneoplastic progression (56). Infectious agents may further shape the gastric microenvironment through distinct inflammatory and oncogenic mechanisms. *H. pylori* promotes chronic inflammation and oncogenic signaling, whereas EBV can modulate immune responses and epigenetic regulation (57). GPL progression is not driven by a single cause but arises from the interplay of inflammation, microbial dysbiosis, and lineage reprogramming, with GIM subtype serving as a key marker for risk stratification.

The gastric microbiota is dominated by Proteobacteria, Firmicutes, Bacteroidetes, Actinobacteria, and Fusobacteria (58–60). In this context, *H. pylori* functions as a “hit-and-run” trigger in gastric carcinogenesis. Its abundance declines with disease progression and may even disappear, while preneoplastic and neoplastic processes continue (61–63). These findings indicate that *H. pylori* is not the sole driver of disease progression. In a randomized controlled trial (RCT) involving 587 infected individuals, persistent inflammation or new onset/progression of atrophy and intestinal metaplasia was observed even after successful eradication (64). These changes were associated with enrichment of oral-derived taxa, including *Peptostreptococcus*, *Granulicatella*, *Streptococcus* and *Parvimonas*, and depletion of potentially protective bacteria such as *Faecalibacterium*. Functional prediction (PICRUSt–KEGG) showed increased amino acid and inositol phosphate metabolism, alongside reduced folate metabolism and NOD-like receptor signaling. Together, these findings suggest that non-*H. pylori* microbiota can sustain persistent gastric inflammation and mucosal progression after *H. pylori* clearance.

During the late stages of the Correa cascade, metagenomic profiling has shown enrichment of Proteobacteria, including potentially pathogenic commensals such as *Proteus* (65, 66). Alterations in the abundance of Proteobacteria taxa, particularly members of Enterobacteriaceae such as *Citrobacter* and *Ochrobactrum*, have been associated with gastric carcinogenesis (67–69).

Clinical and experimental studies indicate a shift from protective to pathogenic microbial profiles during precancerous progression. For example, short-chain fatty acid producers such as *Romboutsia* and *Intestinimonas* are transiently enriched in gastric intraepithelial neoplasia but are depleted in gastric cancer, whereas oral pathogens such as *Parvimonas* and *Prevotella* - 2 accumulate (70). In an N-methyl-N′-nitro-N-nitrosoguanidine (MNNG)-induced rat model, gut dysbiosis was most pronounced at the precancerous lesions of gastric cancer (PLGC) stage, with an elevated Firmicutes/Bacteroidetes ratio, reduced *Akkermansia*, and increased *Escherichia*–Shigella. The PLGC microbiota profile closely resembles that of cancer (71). These findings highlight the dynamic and stage-dependent role of the microbiota in gastric carcinogenesis.

### 3.2 Mucosal immune dysregulation and inflammatory crosstalk

The gastric mucosa senses microbes and damage through innate pattern recognition receptors (PRRs), including Toll-like receptors (TLRs) and NOD-like receptors (NLRs), which detect pathogen-associated molecular patterns (PAMPs) and damage-associated molecular patterns (DAMPs) to initiate inflammatory signaling (72). As early regulators of immune polarization, PRRs influence whether responses favor tolerance or pro-inflammatory activation, thereby shaping gastric mucosal responses under inflammatory conditions (73).

Long-term *H. pylori* colonization shapes gastric mucosal immune recognition through persistent low-level stimulation and sustained inflammatory signaling (74, 75). *H. pylori* dampens acute pro-inflammatory responses by reducing the immunogenicity of key ligands such as lipopolysaccharide (LPS) and flagellin, while shifting immune recognition from surface PRRs toward the intracellular NOD1–NF-κB–IL-8 axis (76–79). This persistent inflammatory state promotes gastric epithelial stress, ROS-mediated DNA damage, and precancerous progression (80).

The gastric mucosa actively maintains local homeostasis through an autonomous innate mucosal immune system. Commensal bacteria induce epithelial IL-7 production, which drives ILC2-derived IL-5 to promote B cell differentiation into IgA^+^ plasma cells. Secreted IgA coats the microbiota, including *H. pylori*, thereby limiting excessive colonization and restraining inflammatory amplification (60, 81). This host defense mechanism is stage-dependent. Asymptomatic *H. pylori* infection is characterized by NKp44^+^ ILC3s and IL-22 signaling, which maintain mucosal barrier integrity and promote epithelial repair. As disease progresses toward chronic inflammation and metaplastic changes, ILC2s accumulate in the gastric mucosa and are strongly associated with spasmolytic polypeptide-expressing metaplasia (SPEM) and tumorigenesis (82, 83). Innate immune dysregulation therefore serves as a key link between microbial dysbiosis and gastric mucosal pathology.

The inflammatory response in GPL is driven by a multicellular network integrating altered PRR signaling, impaired IgA-mediated mucosal homeostasis, neutrophil chemotaxis, and ROS amplification, sustaining a persistent low-grade inflammatory state that promotes aberrant epithelial differentiation and disease progression.

### 3.3 Metabolic alterations and functional consequences

Multi-omics studies indicate that metabolic alterations emerge early in the Correa cascade and evolve with disease progression. Metabolic evolution in GPL is closely associated with inflammatory signaling and microbial dysbiosis, reflecting coupled cellular remodeling. Along the progression from normal mucosa to atrophy and IM, glycerophospholipids decline and the TCA cycle is remodeled, with the metabolic phenotype of IM becoming distinct from that of normal tissue (84). In the CAG stage, gastric microbial composition and metabolic profiles differ from those observed in non-atrophic gastritis. Integrated microbiome and metabolomic analysis identified associations between several bacterial genera and differential metabolites, with enrichment of pathways involving the TCA cycle, retinol metabolism, and amino acid metabolism (85).

Prospective lipidomic analysis have shown that in individuals who progress to gastric cancer, levels of arachidonic acid (FFA 20:4) and α-linolenic acid (FFA 18:3) are significantly reduced and inversely associated with disease progression risk (86). Consistently, IM is characterized by decreased levels of α-linolenic acid, linoleic acid, and palmitic acid, indicating dysregulation of multiple fatty acid metabolic pathways (87). Meanwhile, amino acid metabolism (e.g., tryptophan and arginine metabolism) is characterized by a depletion of several amino acids, accompanied by elevated hydroxylated, dicarboxylic, and unsaturated acylcarnitines, suggesting mitochondrial and peroxisomal dysfunction (88).

GPL is characterized by alterations in energy and lipid metabolism that are closely linked to inflammatory signaling and microbial composition. During the CAG and IM stages, these metabolic pathways remain dynamically altered alongside changes in host–microbiota interactions. These dynamic metabolic changes provide a rationale for interventions targeting microbial dysbiosis and metabolic dysfunction, including postbiotic-based strategies.

### 3.4 Risk stratification and clinical management

GPL represents a continuous pathological spectrum rather than an isolated diagnosis, providing a basis for risk stratification and clinical management. This spectrum encompasses chronic atrophic gastritis characterized by atrophy and/or intestinal metaplasia, along with associated dysplastic changes, including low-grade, high-grade, and indefinite dysplasia. Progression along this spectrum is associated with an increased risk of invasive gastric cancer. Current clinical management centers on risk stratification across this spectrum to identify high-risk individuals and guide *H. pylori* eradication and endoscopic surveillance strategies (4, 89). Risk stratification for GPL is primarily based on two core dimensions: the anatomical extent of lesions (i.e., involvement of both the antrum and corpus) and histopathological staging, most commonly assessed using the Operative Link on Gastritis Assessment (OLGA) and the Operative Link on Gastric Intestinal Metaplasia (OLGIM) systems. High-risk features include extensive intestinal metaplasia, OLGA/OLGIM stage III–IV, incomplete-type intestinal metaplasia, a high endoscopic grade (e.g., high EGGIM scores), a first-degree family history of gastric cancer, and persistent *H. pylori* infection, supporting entry into endoscopic surveillance programs. In contrast, patients with localized lesions, OLGA/OLGIM stage 0–II, and without these high-risk factors have a lower overall risk of gastric cancer and are generally managed more conservatively (5).

Current guidelines establish a risk-stratified management approach for gastric epithelial precancerous conditions. For patients with background atrophy and/or intestinal metaplasia, risk assessment based on OLGA/OLGIM staging and lesion extent determines the need for endoscopic surveillance and appropriate surveillance intervals. In contrast, patients with neoplastic lesions, including indefinite dysplasia (IND), low-grade dysplasia (LGD), and high-grade dysplasia (HGD), require histological reassessment, high-quality endoscopic evaluation, and endoscopic resection or intensified surveillance (6, 17).

Biotic interventions should therefore be considered within, rather than as alternatives to, established risk-stratified management. For dysplastic lesions, endoscopic evaluation and treatment remain the clinical priority, whereas the potential role of biotic interventions in atrophy and intestinal metaplasia remains investigational. A nationwide cohort study from Sweden demonstrated that progression along the Correa cascade is associated not only with increased gastric cancer-related mortality but also with elevated overall and non-cancer mortality risks (90). These findings further highlight the importance of long-term risk assessment in patients with gastric precancerous conditions. The clinical risk stratification, guideline-recommended management, and positioning of biotic interventions across different stages of gastric precancerous progression are summarized in **Table 1**.

**Table 1:** Guideline-based risk stratification and proposed positioning of biotic interventions across gastric precancerous stages.

Stage	Key criteria for risk stratification	Guideline-recommended management	Positioning of biotic interventions	Evidence	Reference
*H. pylori*-associated non-atrophic gastritis	No atrophy or intestinal metaplasia	Test and eradicate *H. pylori*; routine endoscopic surveillance is not required	Potential supportive	Clinical and preclinical evidence	(106, 144)
Localized atrophy or intestinal metaplasia	Mild to moderate atrophy/IM limited to the antrum; OLGA/OLGIM stages 0–II; no family history of gastric cancer or incomplete-type IM	Eradicate *H. pylori*; routine endoscopic surveillance is generally not recommended	Investigational supportive	Limited clinical evidence	(142)
Extensive atrophy or intestinal metaplasia/high-risk stage	At least one of the following: Extensive atrophy or IM involving the corpus and/or OLGA/OLGIM stages III–IV	Eradicate *H. pylori* and perform endoscopic surveillance every 3 years; if family history is present, closer surveillance every 1–2 years may be considered	Investigational supportive	Limited preclinical evidence	(124)
Non-visible dysplasia, including IND, LGD, or HGD	Histological diagnosis indicates dysplasia, but high-quality endoscopy does not identify a visible lesion	Pathology review; immediate high-quality endoscopic reassessment; if no visible lesion is found, repeat endoscopy after 6 months for HGD and after 12 months for LGD/IND	Not applicable	No direct evidence	(5)
Visible lesion/early gastric cancer	Endoscopically visible dysplastic or early cancerous lesion, including differentiated-type pT1a lesions without vascular invasion	Endoscopic resection, preferably ESD; subsequent management based on post-resection pathological risk stratification, such as eCura classification; very-low-risk and low-risk patients may require no additional treatment but should receive scheduled endoscopic follow-up	Not applicable	No direct evidence	(6)
Post-treatment follow-up after gastric cancer treatment	Patients after endoscopic or surgical resection of gastric neoplastic lesions	Endoscopic follow-up interval determined by post-resection pathological risk stratification, such as very-low, low, local, or high risk; follow-up may be scheduled at 3–6 months or annually according to risk level	Potential supportive	Limited clinical evidence	(126, 154)

## 4 Biotic modulation of host-microbiota interactions

As chronic gastritis progresses through gastric atrophy and intestinal metaplasia to dysplasia, microbial, metabolic, immune, and epithelial homeostasis becomes progressively disrupted (91). Accordingly, biotic interventions may act not by targeting isolated pathways but by modulating interconnected microbiota–host regulatory networks. This section examines the evidence for biotic modulation of microbial, metabolic, immune, and barrier pathways across different stages of gastric disease progression, followed by systemic and cross-compartmental effects. An overview of the proposed mechanisms by which biotics may modulate gastric precancerous states is presented in **Figure 2**.

**Figure 2:**
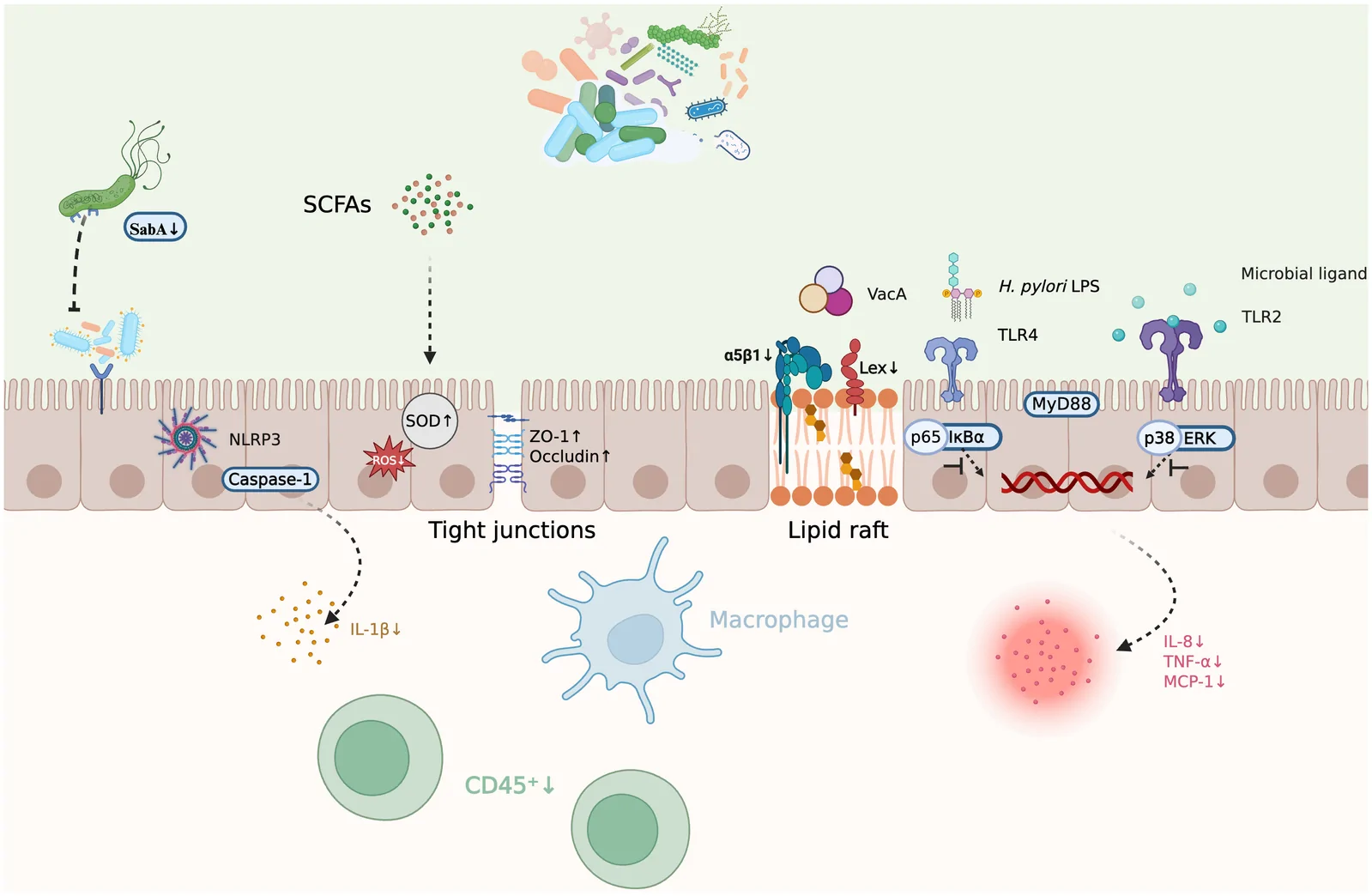
Possible mechanisms for the role of biotics in gastric precancerous states. Gastric precancerous states are characterized by interconnected alterations in microbial ecology, epithelial integrity, immune responses, oxidative stress, and host metabolism. Representative mechanisms illustrated include inhibition of *Helicobacter pylori* adhesion and virulence, modulation of TLR2/TLR4–MyD88, NF-κB, MAPK, and NLRP3 signaling pathways, preservation of epithelial tight junction integrity, attenuation of oxidative stress and inflammatory responses, and metabolic regulation involving postbiotic-derived metabolites such as short-chain fatty acids (SCFAs). These mechanisms are integrated from current experimental and clinical evidence and provide a conceptual framework for understanding microbiome-based nutritional modulation of gastric precancerous states rather than representing a single validated signaling pathway. Abbreviations: SCFAs, short-chain fatty acids; LPS, lipopolysaccharide; TLR, Toll-like receptor; MyD88, myeloid differentiation primary response 88; NF-κB, nuclear factor kappa B; MAPK, mitogen-activated protein kinase; NLRP3, NOD-like receptor family pyrin domain-containing 3; ROS, reactive oxygen species; SOD, superoxide dismutase; ZO-1, zonula occludens-1; MCP-1, monocyte chemoattractant protein-1; IL, interleukin; TNF-α, tumor necrosis factor-alpha; VacA, vacuolating cytotoxin A; SabA, sialic acid-binding adhesin; Leˣ, Lewis x antigen; α5β1, alpha 5 beta 1 integrin. Created with BioRender.com (https://BioRender.com/xax3olh).

### 4.1 Restoration of gastric microbial homeostasis

Disruption of gastric microbial homeostasis may actively contribute to gastric inflammation rather than merely accompany it. Recent experimental evidence showed that a high-nitrate diet induced marked gastric microbial dysbiosis, characterized by reduced microbial diversity, enrichment of potential pathogens, and depletion of beneficial taxa; notably, colonization with *Enterococcus gallinarum*, one of the enriched species, further aggravated gastritis in mice (92). Against this background, biotic interventions may modulate gastric microbial homeostasis through several mechanisms, including suppression of *H. pylori* colonization and modulation of the broader gastric microbial community. Early studies identified several mechanisms through which probiotics interfere with *H. pylori* colonization. *Lactobacillus salivarius* suppresses urease activity and colonization through sustained acid production, whereas soluble or heat-stable factors released by other *Lactobacillus* strains inhibit bacterial growth, reduce adhesion, and downregulate adhesion-related genes (93–95). Direct bacterial interactions may also contribute. *Lactobacillus gasseri* Kx110A1 co-aggregates with *H. pylori* through a sortase A-dependent surface protein system, thereby limiting its access to epithelial binding sites (96). Similar effects have been reported for *L. acidophilus* NCFM and *L. plantarum* Lp-115, which reduced *H. pylori* adhesion and gastric colonization in mice (97). A formulation containing *Lacticaseibacillus paracasei* HP7 also reduced *H. pylori* colonization and virulence-related effects *in vivo*, although the contribution of HP7 cannot be separated from that of the accompanying botanical extracts (98).

Beyond inhibition of *H. pylori* colonization, probiotics also reshape the broader gastric microbiota. More recent evidence showed that combined administration of *Lactiplantibacillus plantarum* HCS03–001 and *Lacticaseibacillus paracasei* HCS17–040 partially restored the gastric microbial alterations induced by *H. pylori*, increasing microbial α-diversity and reducing the elevated Firmicutes/Bacteroidetes ratio in infected mice (99). Notably, mucosal thinning and a reduction in gastric glands were also observed in the infected mice, suggesting possible early atrophic changes beyond inflammatory injury, although more systematic histological characterization would be required to establish gastric atrophy. *Lactobacillus plantarum* ZDY2013 and ZFM4 partially restore phylum-level dysbiosis and preserve microbial diversity in *H. pylori* infection models (98, 100). More targeted effects have been observed with *Lactiplantibacillus plantarum* ZJ316 and *Lacticaseibacillus paracasei* ZFM54. ZJ316 suppresses *H. pylori* adhesion, downregulates virulence-associated gene expression, and selectively enriches short-chain fatty acid–producing taxa such as *LigiLactobacillus* and *Akkermansia*. In contrast, ZFM54 broadly reshapes the gastric microbiota, increasing *Firmicutes* and *Actinobacteriota* while reducing Campylobacterota and Proteobacteria (101–103).

Recent studies further expanded our understanding of the mechanisms underlying probiotic intervention in *H. pylori*–associated dysbiosis. *Lactococcus garvieae* LG3092, through secretion of the bacteriocin Garvicin Q, selectively remodels the gastric microbiota and reduces pro-inflammatory cytokines (IL-1β, IL-6, and TNF-α) without directly eradicating *H. pylori*, suggesting that microbe-derived effector molecules may contribute to the restoration of gastric homeostasis (104). Clinical findings, however, indicate that suppression of *H. pylori* does not necessarily coincide with broader microbiota restoration. In a randomized, double-blind, placebo-controlled trial, *Lactobacillus crispatus* FSCDJY67L3 reduced *H. pylori* load and gastrointestinal symptom scores, but did not significantly alter gut microbial diversity or overall community composition (105). In a recent randomized, double-blind, placebo-controlled trial, co-administration of *Lactiplantibacillus plantarum* MH-301 during *H. pylori* eradication therapy reduced gastrointestinal adverse events and changed the relative abundance of several gut bacterial genera. However, it did not significantly improve gut microbial diversity or the *H. pylori* eradication rate (106).

In addition to *H. pylori*, the oral pathogen *Fusobacterium nucleatum* has also been implicated in gastric carcinogenesis. Recent experimental evidence showed that *F. nucleatum* exhibits marked acid tolerance, which is associated with the regulation of erucic acid [C22:1(n9)] synthesis by FnFabM. This property facilitates its survival in the acidic gastric environment and passage through the upper gastrointestinal tract (107). *F. nucleatum* is enriched in gastric cancer tissues with lymph node metastasis and promotes tumor progression by activating the IL-17/NF-κB/RelB axis and inducing immune evasion (108). Evidence from colorectal cancer models shows that *Bifidobacterium animalis* counteracts *F. nucleatum*-induced lipid metabolic reprogramming, inflammatory amplification, and barrier disruption (109). These findings suggest that mechanisms involving *F. nucleatum* may also operate in gastric precancerous conditions, although direct evidence in the gastric context remains limited.

Most evidence for the microbiota-modulating effects of biotic interventions comes from *H. pylori*-associated gastritis, where reductions in bacterial colonization and partial restoration of gastric microbial composition have been reported. Gastric dysbiosis, however, extends beyond this early stage, with progressive microbial restructuring reported along the Correa cascade, including gastric atrophy, intestinal metaplasia, and more advanced lesions (110). This persistent dysbiosis suggests that microbial homeostasis may remain relevant beyond the gastritis stage, but its therapeutic significance is uncertain. Direct evidence that biotic interventions improve established atrophy, intestinal metaplasia, or dysplasia through restoration of the gastric microbiota remains limited. Thus, microbiota modulation is currently best supported as a mechanism of biotic action in gastritis, whereas its role in more advanced precancerous lesions remains to be established.

### 4.2 Modulation of metabolic homeostasis and postbiotic signaling

Progression of gastric mucosal lesions is accompanied by metabolic changes involving lipids, amino acids, and microbially derived metabolites. Biotic interventions may modulate these metabolic features via microbial metabolites or host pathways (87, 111, 112). However, evidence for these effects varies by disease stage and between the gastric and intestinal environments, and mechanisms established in the intestine may not translate directly to the gastric mucosa.

Butyrate is one of the best-characterized microbial metabolites and can regulate host metabolism and immune responses through HDAC inhibition, G protein-coupled receptors, and metabolic signaling pathways such as AMPK and PPAR-γ (113, 114). In intestinal and systemic models, these effects include modulation of tumor-cell metabolism, regulatory T-cell differentiation, and CD8^+^ T-cell function (115–120). However, most of these mechanisms were established in colonic or systemic settings rather than in the gastric mucosa, and their relevance to gastric precancerous lesions therefore remains uncertain.

Direct evidence that biotic interventions exert metabolic effects in gastric disease remains limited, with most data coming from *H. pylori*-associated models. *Heyndrickxia coagulans* BHE26 produced substantial amounts of lactic and acetic acids, and neutralization of its fermentation supernatant markedly reduced its inhibitory activity against *H. pylori*, supporting a functional role for secreted organic acids in its antibacterial effect (121). In a recent metabolomic study, combined administration of *Clostridium butyricum* and *Bacillus coagulans* to *H. pylori*-infected mice partially reversed alterations in fecal and serum metabolites involving linoleic and arachidonic acid metabolism, glycerophospholipid metabolism, tryptophan metabolism, and primary bile acid biosynthesis (122). Additional evidence comes from the gastric isolate *Lactobacillus paragasseri* LPG-9, which, together with other gastric *Lactobacillus* isolates, produced several fatty acids *in vitro*, including propionic and butyric acids. In *H. pylori*-infected mice, LPG-9 also reduced gastric inflammation and partially restored predicted microbiota-associated amino acid metabolic alterations (123). Together, these findings suggest that probiotics can generate bioactive metabolites and modulate metabolic features associated with *H. pylori* infection, although whether specific microbial metabolites directly mediate gastric mucosal protection remains unclear.

Evidence linking these metabolic changes to more advanced gastric lesions is even weaker. In INS-GAS mice, supplementation with *Lactobacillus salivarius* Chen-08 and *Lactobacillus rhamnosus* LGG-18 reduced inflammation, atrophy, intestinal metaplasia, and dysplasia while increasing the abundance of several SCFA-producing taxa, including *Bacteroides*, *Alloprevotella*, and *Oscillibacter* (124). However, SCFA concentrations were not measured, and a causal role for SCFA signaling was not established. Gastric-specific evidence from direct exposure studies is also limited. Sodium butyrate showed antiproliferative effects and altered proteomic profiles in AGS gastric adenocarcinoma cells (125), whereas an RCT of *C. butyricum* CGMCC0313.1 after gastrectomy increased fecal SCFAs and was accompanied by reduced systemic inflammation and improved immune indices (126). The former represents a gastric cancer cell model, whereas the latter primarily reflects intestinal and systemic effects rather than local gastric SCFA signaling.

Nevertheless, intestinal SCFA-mediated immunomodulation may not translate directly to the gastric microenvironment. Although gastric-derived probiotic strains can produce SCFAs *in vitro*, whether these metabolites reach biologically relevant concentrations *in vivo* and mediate mucosal effects remains unclear. Consistent with this uncertainty, gastric fluid SCFA levels were substantially lower than serum levels and did not differ significantly between normal and gastric cancer mice despite marked differences in gastric microbial composition (127). Moreover, the immunomodulatory effects of butyrate are concentration- and cell-context-dependent, ranging from induction of colonic regulatory T cells to enhanced cytotoxic CD8^+^ T-cell responses (119, 120). Thus, SCFAs may influence gastric disease through intestinal and systemic metabolic–immune pathways, whereas a comparable local SCFA–immune axis in the gastric mucosa has not been established.

Overall, direct evidence for biotic modulation of metabolic pathways is concentrated in *H. pylori*-associated gastritis. Evidence is considerably weaker in gastric atrophy, intestinal metaplasia, and dysplasia. In these stages, shifts in SCFA-producing taxa have been reported alongside histological improvement, but whether microbial metabolites mediate these effects remains unclear. Future studies using isotope tracing alongside multi-omics are needed to distinguish locally produced from gut-derived microbial metabolites and determine their contribution to gastric mucosal responses across disease stages.

### 4.3 Immunomodulation and barrier function restoration

#### 4.3.1 Modulation of immune and inflammatory signaling

PRR-mediated sensing is a key mechanism through which the gastric mucosa detects microbial signals. However, compared with the intestinal system, direct evidence for probiotic-mediated PRR signaling remains limited, partly due to the stomach’s acidic environment, low microbial load, and the predominant focus on *H. pylori* (83, 128). In gastric epithelial cells, *Lactobacillus bulgaricus* suppressed *H. pylori* LPS-induced TLR4–NF-κB signaling by inhibiting IκBα degradation and p65 nuclear translocation (129). A related effect has been reported for the probiotic formulation BIFICO, containing *Enterococcus faecalis*, *Bifidobacterium longum*, and *Lactobacillus acidophilus*, which reduced TLR2/TLR4/MyD88 signaling together with downstream NF-κB and MAPK activation in experimental *H. pylori* gastritis (130).

Probiotics may also interfere with *H. pylori* virulence factor delivery and activity. *Lactobacillus gasseri* Kx110A1 reduced CagA delivery and the associated hummingbird phenotype, and suppressed TNF-α and IL-6 production by downregulating ADAM17 in macrophages (130, 131). *Lactobacillus rhamnosus* JB3 similarly interfered with CagA translocation and VacA internalization by altering lipid raft-associated host receptors, including Lewis x and α5β1 integrin, with subsequent suppression of p38/ATF-2 signaling (132). Probiotic-mediated regulation of inflammatory signaling also involves transcription factors such as NF-κB and AP-1, including its c-Jun component (133, 134).

Postbiotics derived from *Streptococcus thermophilus* and *Lactobacillus casei* also suppressed NF-κB and pro-inflammatory cytokine expression in *H. pylori*-challenged AGS cells (135). Beyond these canonical inflammatory pathways, biotic interventions may also modulate inflammasome activation and inflammatory cell death. The gastric-derived probiotic *Weizmannia coagulans* BCF-01 inhibited TLR4–MyD88–NF-κB signaling and NLRP3 inflammasome-mediated pyroptosis in *H. pylori*-infected gastric epithelial cells, accompanied by reduced inflammatory mediator release and pyroptotic cell death (136).

In a non–*H. pylori*-associated multifactorial gastritis model, *Clostridium butyricum* and a synbiotic intervention reduced gastric IL-1α, IL-6, and TGF-β1 expression in rats and downregulated an inflammation-to-cancer transition gene signature (137). Notably, although this signature was derived from human datasets spanning non-atrophic gastritis, chronic atrophic gastritis, and intestinal metaplasia, the interventions were tested only in rats with gastritis. Thus, the apparent stage specificity of these immunomodulatory effects may partly reflect the distribution of available evidence rather than a biological restriction to gastritis. Most mechanistic studies have used *H. pylori*-associated gastritis models, whereas established atrophy, intestinal metaplasia, and dysplasia have rarely been examined directly. Whether biotics can still modify inflammatory signaling once persistent epithelial remodeling has developed remains unresolved.

#### 4.3.2 Restoration of barrier integrity and epithelial function

Early mucosal injury in the Correa cascade involves disruption of tight junction integrity, oxidative stress, and dysregulated epithelial proliferation (138, 139). The gastric epithelium also retains substantial plasticity in response to injury. Lineage-tracing studies have shown that differentiated chief cells can give rise to SPEM following acute mucosal injury and contribute to subsequent epithelial lineage remodeling (10).

Recent studies of gastric-derived probiotic strains provide evidence for modulation of barrier integrity and epithelial injury. *Weizmannia coagulans* BCF-01 increased ZO-1 and occludin expression while reducing epithelial apoptosis, whereas *L. salivarius* NCUH062003 restored tight junction protein expression, corrected Ki-67/β-catenin dysregulation, and increased superoxide dismutase (SOD) activity (136, 140). *Lactobacillus paragasseri* LPG-9 similarly promoted gastric mucosal barrier restoration in *H. pylori*-infected mice (123). Together, these findings suggest that biotic interventions can modulate multiple features of epithelial injury, including barrier disruption, oxidative stress, and proliferative dysregulation.

Beyond barrier restoration, certain probiotic strains may also modulate epithelial phenotypes associated with disease progression. *Lactobacillus gasseri* BIO6369 and *L. rhamnosus* BIO5326 suppressed EMT-related phenotypes by downregulating Snail and Zeb1, preserving ZO-1 localization, and reducing hummingbird morphology and tumorosphere formation (141). More recently, *L. acidophilus* and *Bifidobacterium lactis* suppressed *H. pylori*-induced miR-185–Wnt/β-catenin–COX-2 signaling in gastric epithelial cells (142). In a six-month RCT, probiotic supplementation after *H. pylori* eradication was associated with a higher rate of histological intestinal metaplasia regression than the control treatment. However, the miR-185–Wnt/β-catenin–COX-2 mechanism was primarily established in the accompanying *in vitro* experiments rather than demonstrated as the mediator of IM regression. The available preclinical evidence for biotic interventions in gastric precancerous states is summarized in **Table 2**.

**Table 2:** Preclinical evidence for biotic interventions in gastric precancerous states.

Disease stage	Biotic intervention and strain	Model	Dose and duration	Histological and other key outcomes	Reference
—	*Lactobacillus gasseri* BIO6369 and *Lacticaseibacillus rhamnosus* BIO5326	*H. pylori*-infected AGS cells	Probiotics: MOI 10; *H. pylori*: MOI 50; 24 h	Suppressed EMT and preserved epithelial barrier integrity	(141)
—	*Lactobacillus gasseri* Kx110 A1	*H. pylori*-infected and CagA-transfected AGS cells	MOI 50 (*H. pylori* infection) or 100 (CagA transfection); 16 h or 5 h, respectively	Reduced CagA activity and associated morphological changes	(130)
Gastritis, gastric atrophy, intestinal metaplasia, and dysplasia	*Lactobacillus rhamnosus* LGG-18 + *Lactobacillus salivarius* Chen-08	*H. pylori*-infected INS-GAS mice	1 × 10^9^ CFU/mL in drinking water; 12 weeks	Reduced gastric inflammation, atrophy, intestinal metaplasia, and dysplasia; reduced epithelial proliferation and altered gastric and gut microbiota	(124)
*H. pylori*-associated gastritis	*Weizmannia coagulans* BCF-01	*H. pylori*-infected C57BL/6 mice	3 × 10^9^ CFU/day; 7 days	Reduced gastric mucosal injury and inflammation; reduced *H. pylori* infection and improved gastric microbiota and mucosal barrier integrity	(136)
*H. pylori*-associated gastritis with glandular atrophy	*Lactococcus garvieae* LG3092	*H. pylori*-infected C57BL/6 mice	5 × 10^8^ CFU/day; 6 days	Reduced glandular atrophy and mucosal injury; reduced *H. pylori* colonization and gastric inflammation	(104)
*H. pylori*-associated gastritis	*Lactiplantibacillus plantarum* HCS03-001 + *Lacticaseibacillus paracasei* HCS17-040	*H. pylori*-infected C57BL/6 mice; preventive and therapeutic models	1 × 10^9^ CFU/mouse; prevention: 2 weeks; treatment: 7 days	Reduced gastric inflammatory cell infiltration; reduced *H. pylori* virulence; restored gastric microbial composition	(99)
*H. pylori*-associated gastritis	Postbiotics derived from *Streptococcus thermophilus* and *Lactobacillus* casei	*H. pylori*-infected AGS cells and Wistar rats	12.5 μg/mL *in vitro*; 3.75 mL/rat/day *in vivo*; 96 h and 10 days, respectively	Reduced gastric inflammation and *H. pylori* burden; inhibited inflammatory signaling	(135)
*H. pylori*-associated gastritis	*Heyndrickxia coagulans* BHE26 and its fermentation supernatant	*H. pylori* SS1-infected C57BL/6J mice; preventive and therapeutic models	BHE26: 5 × 10^8^ CFU/mL, 0.2 mL/day; supernatant: 0.2 mL/day; 3–4 weeks	Preserved gastric mucosal architecture and reduced inflammation; reduced *H. pylori* colonization and gastric dysbiosis	(121)

### 4.4 Systemic and extra-gastric effects

During *H. pylori* infection, probiotic interventions can influence not only the gastric mucosa but also intestinal and systemic compartments. In infected mice, multi-strain probiotic supplementation reduced gastric inflammation while altering circulating metabolic and immune parameters (143). Human studies have also reported changes outside the gastric mucosa. In an RCT, probiotic supplementation during *H. pylori* eradication therapy was associated with changes in microbial communities across the gastric mucosa, intestine, and oral cavity (144). Other RCTs have reported effects beyond the stomach. *Lacticaseibacillus rhamnosus* LRa05 supplementation was associated with changes in gut microbiota and circulating inflammatory markers (145), while *Lactiplantibacillus plantarum* MH-301 altered both gut and vaginal microbial communities (106). By contrast, *Lacticaseibacillus paracasei* HP7 reduced *H. pylori* activity and serum TNF-α without substantially altering overall gut microbial diversity (146). In *H. pylori*-infected rats, *L. rhamnosus* and *L. plantarum* also improved anxiety- and depression-like behaviors together with changes in cortical inflammatory, oxidative, neurotrophic, and serotonergic markers (147). However, these effects have not been consistent across interventions. A compound probiotic regimen better preserved gut microbial stability than bismuth quadruple therapy in one RCT (148), whereas *L. reuteri* supplementation did not significantly prevent antibiotic-associated gut dysbiosis in another (149). Together, these studies suggest that probiotic interventions can affect multiple body sites, although whether intestinal or systemic changes mediate gastric mucosal outcomes remains unclear.

Despite these observations, a direct causal gut–gastric–immune axis has not been established. Unlike the gut–liver axis, which is linked by portal circulation and bile acid metabolism, or the microbiota–gut–brain axis, which involves neural, endocrine, and immune routes, communication between the intestinal microbiota and gastric mucosa lacks a clearly defined route (150–152). Gut-derived metabolites, immune mediators, and microbial products may contribute to systemic regulation, but their exposure and functional activity within the gastric mucosa remain poorly characterized. This limitation is particularly relevant to SCFAs, whose local gastric effects cannot be inferred directly from intestinal or circulating concentrations, as noted earlier. Thus, current evidence is better interpreted as cross-compartmental associations and systemic crosstalk rather than proof of a directional gut-to-gastric signaling axis.

Most clinical evidence for such cross-compartmental effects comes from patients undergoing treatment for *H. pylori* infection, predominantly at the gastritis stage. Whether similar cross-compartmental effects occur in gastric atrophy, intestinal metaplasia, or dysplasia remains unclear. In addition, safety should be considered in clinical translation. Although pharmacovigilance data suggest that probiotic-associated adverse events are generally uncommon, clinically relevant risks may occur in susceptible populations (153). Future studies should combine longitudinal sampling of multiple compartments with histological endpoints to determine whether these changes are associated with, precede, or contribute to gastric mucosal improvement across disease stages. The available clinical evidence for biotic interventions in gastric precancerous states is summarized in **Table 3**.

**Table 3:** Randomized controlled trials of biotic interventions in gastric precancerous states.

Disease stage	Biotic intervention and strain	Population	Dose and duration	Histological and other key outcomes	Reference
*H. pylori*-associated chronic gastritis	*Bifidobacterium* infantis CGMCC0460.1, *Lactobacillus acidophilus* CGMCC0460.2, Enterococcus faecalis CGMCC0460.3, and Bacillus cereus CGMCC0460.4	276 treatment-naïve *H. pylori*-positive adults with chronic gastritis	9 tablets once daily; 4 weeks	Histology not assessed; reduced gastrointestinal adverse events and microbiota perturbations without significantly improving *H. pylori* eradication	(144)
*H. pylori*-associated gastritis	*Lacticaseibacillus rhamnosus* R0011 + *Lactobacillus helveticus* R0052	90 patients with *H. pylori* gastritis	4 × 10^9^ CFU/capsule, 1 capsule twice daily; 42 days	Histology not assessed; reduced gastrointestinal adverse events and symptom scores; increased *H. pylori* eradication in PP but not ITT analysis	(155)
*H. pylori* infection	*Enterococcus faecium* + *Bacillus subtilis* (Medilac-S^®^)	270 *H. pylori*-positive adults	E. faecium 4.5 × 10^8^ CFU + B. subtilis 5.0 × 10^7^ CFU, 1 tablet three times daily; 4 weeks	Histology not assessed; did not significantly improve *H. pylori* eradication, recrudescence, or dyspeptic symptoms	(156)
*H. pylori* infection	*Lacticaseibacillus rhamnosus* LRa05	72 *H. pylori*-positive adults	1 × 10¹^0^ CFU/day; 4 weeks	Histology not assessed; did not significantly improve *H. pylori* eradication; reduced gastrointestinal symptoms and treatment-associated gut microbiota alterations	(145)
*H. pylori*-associated chronic gastritis	*Lactiplantibacillus plantarum* MH-301	257 women with *H. pylori* infection and chronic gastritis	>1 × 10^9^ CFU/day; 14 days	Histology not assessed; reduced gastrointestinal adverse events; did not significantly improve *H. pylori* eradication or gut microbial diversity	(106)
Intestinal metaplasia	*Lactobacillus acidophilus* LA-5^®^, *Bifidobacterium* lactis BB-12^®^, and *L. acidophilus* La-14	58 patients with histologically confirmed gastric IM after successful *H. pylori* eradication	≥5 × 10^9^ live organisms/pack, twice daily; 6 months	Increased histological IM regression (ITT: 37.5% vs 11.5%; PP: 46.2% vs 17.6%); experimental analysis suggested modulation of miR-185–Wnt/β-catenin–COX-2 signaling	(142)
*H. pylori* infection	*Lacticaseibacillus paracasei* HP7	78 *H. pylori*-positive adults with functional dyspepsia	1.5 × 10^9^ CFU/day or 5.0 × 10^9^ CFU/day; 8 weeks	Histology not assessed; high-dose HP7 reduced *H. pylori* activity; both doses increased the pepsinogen I/II ratio and reduced systemic inflammation	(146)

## 5 Conclusion

GPL should be understood as a dynamic process shaped by the interplay of microbial dysbiosis, metabolic alterations, and immune and barrier dysfunction, rather than as the consequence of a single pathogen or signaling pathway. Thus, biotic interventions may influence interconnected microbial, metabolic, immune, and barrier processes, although the strength of evidence differs substantially across disease stages.

However, available evidence is limited by the lack of prospective studies with histological or long-term risk endpoints, as well as substantial heterogeneity in strains, formulations, baseline microbiota, and concomitant therapies. In addition, most mechanistic insights are derived from *H. pylori* infection or gastritis models, which do not fully capture the persistent glandular and epithelial remodeling characteristic of more advanced precancerous lesions. Further work should shift from assessing whether these interventions are effective to determining for whom, when, and in what form they may be beneficial.

Overall, GPL is better understood as a dynamic risk state rather than a fixed pathological endpoint. Within this framework, biotic interventions may contribute to risk modification and homeostatic support, but should not be considered substitutes for *H. pylori* eradication, endoscopic surveillance, or treatment of established neoplastic lesions.
